# Nephrometry scoring systems: their importance for the planning of nephron-sparing surgery and the relationships among them

**DOI:** 10.1590/0100-3984.2021.0166

**Published:** 2022

**Authors:** Victor Dubeux, José Fernando Cardona Zanier, Carolina Gianella Cobo Chantong, Fabricio Carrerette, Pedro Nicolau Gabrich, Ronaldo Damião

**Affiliations:** 1 Hospital Universitário Pedro Ernesto da Universidade do Estado do Rio de Janeiro (HUPE-UERJ), Rio de Janeiro, RJ, Brazil.

**Keywords:** Radiology/standards, Urology/standards, Nephrectomy/methods, Kidney neoplasms/diagnosis, Magnetic resonance imaging, Tomography, X-ray computed, Radiologia/normas, Urologia/normas, Nefrectomia/métodos, Neoplasias renais/diagnóstico, Ressonância magnética, Tomografia computadorizada

## Abstract

In recent years, the development of new imaging techniques and scoring systems have improved the diagnosis and management of small renal masses. Imaging-based nephrometry scoring systems play an interesting role in the planning of nephron-sparing surgery, providing surgeons with the information necessary to determine the complexity of the renal mass, to deliver the appropriate postoperative care, and to predict adverse outcomes. The aim of this study was to review nephrometry scoring systems, evaluating their characteristics and the relationships among them. The urology and radiology communities should decide which nephrometry scoring system will prevail and be used in daily practice.

## Abstract (pt)

O diagnóstico e o manejo das pequenas massas renais têm sido aprimorados nos últimos anos com o desenvolvimento de técnicas de imagem e escores que desempenham papel interessante no planejamento da nefrectomia parcial, fornecendo informações importantes ao cirurgião para determinar o tipo de tratamento em relação a complexidade da massa renal, cuidados pós-operatórios e previsão de complicações após as cirurgias. O objetivo deste estudo é revisar os escores de nefrometria, suas características e relações entre eles no cenário da cirurgia. Os serviços de urologia e radiologia devem decidir qual é o melhor escore de nefrometria para ser utilizado na prática diária.

## INTRODUCTION

Over the last few decades, there has been a shift toward performing minimally invasive nephron-sparing surgery (NSS) for small renal masses (SRMs) that are clinically localized^(1)^. The diagnosis and management of SRMs have changed with remarkable rapidity because of the wide dissemination of multiple imaging techniques, which has had a direct impact on the clinical decision-making process related to the choice of treatment^(2,3)^.

Although the incidence of renal cell carcinoma (RCC) has been increasing, survival has improved substantially. The newfound popularity of NSS techniques and minimally invasive ablative procedures is probably attributable to the fact that active surveillance has made the incidental diagnosis of small indolent cancers more frequent^(4,5)^. Currently, the main treatment is NSS, which may be performed as an open, conventional laparoscopic, or robotic procedure^(6)^. Regardless of the approach, NSS requires surgical team training, has a long learning curve, and is associated with a significant rate of complications^(6-8)^. The main issue in this situation is that the decision making involves the radiological aspect of the SRM, the experience of the surgeon (one surgeon might decide that a case is suitable for treatment with a minimally invasive technique, whereas another might consider the same case too risky and recommend an ablative approach), and the expectations of the patient.

Nephrometry scoring systems play an important role by providing information that allows the surgical team to determine the complexity of each renal mass in candidates for NSS^(9)^. The aim of this study was to review the principal nephrometry scoring systems, their characteristics, and the relationships among them.

## NEPHROMETRY SCORING SYSTEMS

The aim of nephrometry scoring systems is to allow the surgical specimen to be excised intact, with tumor-free surgical margins and short warm ischemia time, without significant complications. Nephrometry scoring systems constitute an important tool to aid surgical teams and patients, allowing a specific type of NSS to be indicated, as well as predicting preoperative, intraoperative, and postoperative complications^(10)^.

Pre-NSS images are obtained by magnetic resonance imaging (MRI) or computed tomography (CT). In such images, the most important aspects of the tumor are size, penetration through the renal parenchyma, distance to the collecting system, location in relation to the polar lines, and the various characteristics of the renal sinus.

The main nephrometry scoring systems available are as follows: the **R**adius, **E**xophytic properties, **N**earness to the collecting system or sinus, **A**nterior/posterior face, and **L**ocation relative to the polar lines (RENAL) nephrometry score, proposed by Kutikov et al.^(11)^; the Preoperative Aspects and Dimensions Used for an Anatomical (PADUA) classification system, proposed by Ficarra et al.^(12)^; and the centrality index (C index), proposed by Simmons et al.^(13)^. In 2014, Hakky et al.^(14)^ proposed the zonal **Ne**arness to the collecting system, **Ph**ysical location of the tumor in the kidney, **R**adius of the tumor, and **O**rganization of the tumor (NePhRO) scoring system as an improvement on the RENAL nephrometry score.

### RENAL nephrometry score

**Figure 1:**
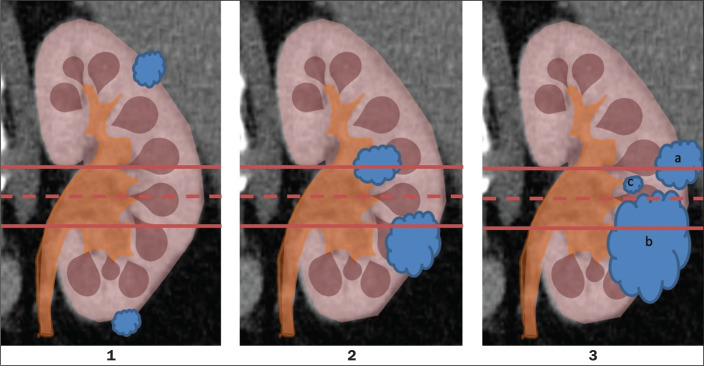
RENAL nephrometry score based on a CT scan. The numbers 1 to 3 represent points assigned to each category of tumor. The polar lines (solid lines) are determined in a sagittal view of the kidney. a, > 50% of the mass is above the upper polar line or below the lower polar line; b, the mass crosses the axial renal midline; c, the mass is entirely between the polar lines.

Source: Adapted from Kutikov et al.^(11)^.

As illustrated and detailed in Figure 1 and Table 1, respectively, the RENAL nephrometry score attributes points ranging from one to three for each of five components^(11)^: the radius of the mass; the exophytic/endophytic properties of the mass (i.e., the degree of parenchymal penetration); the nearness of the mass to the renal collecting system or sinus; the position of the mass (anterior, posterior, or indeterminate), and the location of the mass in relation to the polar lines. One advantage of this score is that it is easily applied. The fact that it evaluates only those five components makes it easy for urologists and radiologists to use.

**Table 1:** Features employed in order to calculate the RENAL score.

Feature	Points		
	1	2	3
Radius (maximum diameter)	≤ 4 cm	> 4 but < 7 cm	≥ 7 cm
Exophytic proportion	≥ 50%	< 50%	0% (100% endophytic)
Nearness of the tumor to the renal collecting system or sinus	≥ 7 mm	> 4 but < 7 mm	≤ 4 mm
Anterior or posterior face	No points given; mass assigned a descriptor of anterior, posterior, or indeterminate		
Location relative to the polar lines	Entirely above the upper polar line or entirely below the lower polar line	Mass crosses a polar line	> 50% of the mass is above the upper polar line or below the lower polar line; the mass crosses the axial renal midline; or the mass is entirely between the polar lines

Source: Adapted from Kutikov et al.^(11)^.

**Figure 2:**
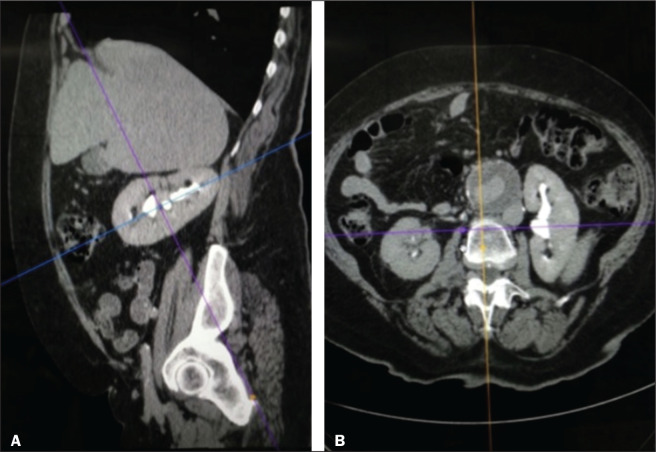
Sagittal and axial CT reconstructions of the kidney (A and B, respectively), based on the main axis of the organ. Note how, in the sagittal view, the kidney is rotated in relation to the spine.

One pitfall of the RENAL nephrometry score is that the location of a mass in relation to the kidney is usually defined by drawing the polar lines, in a coronal view. If the reconstruction of the image of the kidney is based on the spinal axis, the localization of the mass in relation to the kidney could be inaccurate. Typically, the kidneys are angled in relation to the spinal axis, the upper pole being more medial and posterior than the lower pole. Therefore, such reconstructions should be based on the renal axis, in a sagittal view. A tumor initially thought to be crossing the polar lines could, when appropriately evaluated, be found to be in a different location (Figure 2).

Another issue related to the RENAL nephrometry score and other nephrometry scoring systems is the assessment of the nearness of a tumor to the renal collecting system. When based on CT scans, that assessment should be performed in the delayed phase of a contrast-enhanced examination. When MRI is employed, urine is used as the contrast medium to measure the distance of the mass from the collecting system. The choice of imaging modality could also affect the total score in a nephrometry scoring system^(15)^.

Interobserver variation has been studied for the RENAL nephrometry score. In a retrospective study conducted by Vilaseca et al.^(16)^, two independent radiologists applied the RENAL nephrometry score to evaluate 46 patients with renal masses who had undergone imaging examinations between 2008 and 2012. The interobserver agreement was calculated for the total score and for each component score. The authors found that the agreement for each RENAL score component was 98%, 80%, 100%, 89%, and 85%, respectively-nearness, radius, and total score showing the best agreement-whereas it was 89% for a hilar location and 93% for the total score. For the cases in which there was disagreement in terms of the total score, no major implications for surgical planning were observed.

Cost et al.^(17)^ studied the RENAL nephrometry score in children, adolescents, and young adults, the scores being calculated by two reviewers who were blinded to the clinical data. The authors compared tumor characteristics and oncologic outcomes among the masses classified as low-, moderate-, and high-complexity by the RENAL nephrometry score. Of 69 kidneys analyzed, 76% contained high-complexity masses. Low- and moderate-complexity masses, which were more common among the older patients, were typically managed with NSS, and most of them were RCCs or other non-Wilms’ renal tumors. The authors also found that the RENAL nephrometry score did not correlate significantly with blood loss, operative time, blood transfusion, positive surgical margins, or tumor rupture, showing that the score is useful for evaluating the complexity of RCC and other masses in older children.

Reyes et al.^(18)^ studied the RENAL nephrometry score as a means of evaluating the complexity of renal masses to determine their suitability for management by ablative techniques. In that study, thermal ablation was performed in 39 patients with SRMs. Using the RENAL nephrometry score, the authors classified the risk, for each mass, as low (≤ 6), intermediate (7-9), or high (≥ 10). Among the 39 patients evaluated in that study, recurrence was observed in six (15.4%), the masses having been classified as being of moderate complexity in five and of low complexity in one. Complications occurred in only one case, in which the mass had been classified as being of moderate complexity.

Another ablative technique used for the management of SRMs is laparoscopic cryoablation. Okhunov et al.^(19)^ reported 210 cases of patients undergoing cryoablation at three different facilities, stratified by risk using the RENAL nephrometry score. The mean score was 6.1 (range, 4-12). There was a significant difference in the rate of complications, which was 0% among the patients in whom the SRM was classified as being of low complexity (n = 47), 35% among those in whom it was classified as being of moderate complexity (n = 23), and 100% among those in whom it was classified as being of high complexity (n = 7). In a multivariate analysis, the authors found that the RENAL nephrometry score was independently associated with the risk of postoperative complications.

In a prospective study of 150 patients with SRMs submitted to laparoscopic NSS between 2015 and 2018, Dubeux et al.^(20)^ used the RENAL nephrometry score to classify the risk as high (≥ 7) or low (≤ 6). Among the 89 patients with low-risk SRMs, adverse events were observed in 23 (25.8%), compared with 27 (44.3%) of the 61 patients with high-risk SRMs (*p* < 0.05). When the authors evaluated each component of the score, they concluded that none of the components, in isolation, was able to predict adverse events. However, they also concluded that the total RENAL nephrometry score is useful for predicting adverse outcomes in patients with high-risk SRMs.

### PADUA score

**Figure 3:**
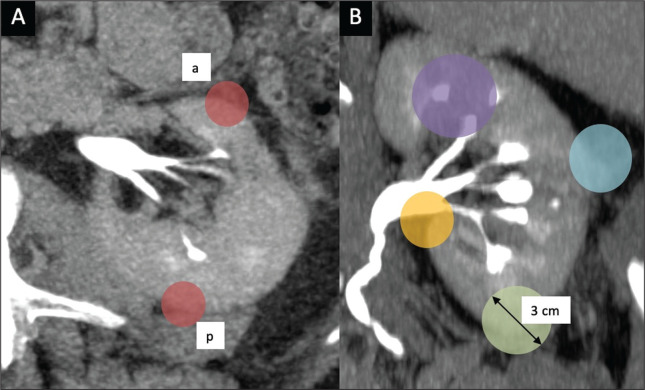
Examples of anatomical features evaluated with the PADUA nephrometry scoring system on axial and coronal CT scans (A and B, respectively). Simulated endophytic mass (purple circle in B), located in the upper pole and in contact with the renal sinus. Another simulated mass (blue circle in B), near the renal rim (lateral), > 50% exophytic, and not in contact with the renal colleting system or renal sinus. Another simulated mass (yellow circle in B), near the renal hilum, in contact with the collecting system and sinus. Yet another simulated mass (green circle in B), located in the lower pole, < 50% exophytic, not in contact with the collecting system or sinus, and 3 cm in diameter. a, anterior; p, posterior.

Source: Adapted from Ficarra et al.^(12)^.

Ficarra et al.^(12)^ proposed the PADUA classification system, which evaluates seven anatomical features of a renal mass: anterior or posterior face; longitudinal (polar) location; margin location (renal rim); relationship with the renal sinus; relationship with the renal collecting system; percentage extending into the parenchyma (exophytic or endophytic); and maximum diameter. Like the RENAL nephrometry score, each aspect is graded in points, as illustrated in Figure 3 and detailed in Table 2. A mass is given one point if it is located entirely above or below the polar lines, two points if 50% of it is above the upper polar line or below the lower polar line, and three points if it is located in the renal sinus. The renal sinus has been defined as the area surrounded by parenchyma, filled by the renal pelvis, fat, and blood vessels^(21)^. The PADUA classification system stratifies renal masses into two groups: with and without involvement of the renal sinus. For the relationship with the renal collecting system, a mass is given one point if it is not in proximity to the system, two points if it is, and three points if it has invaded or distorted the system. If more than 50% of the mass is exophytic, it is given one point, whereas it is given two points if more than 50% is endophytic. Based on its diameter along the longest axis, a mass is given one point (≤ 4 cm), two points (4.1-7.0 cm), or three points (> 7 cm).

**Table 2:** Anatomical features employed in order to calculate the PADUA score.

Feature	Points^*^		
	1	2	3
Anterior or posterior face	No points given; mass assigned a descriptor of anterior or posterior		
Longitudinal (polar) location	Upper	Lower	-
Exophytic rate	≥ 50%	< 50%	Endophytic
Renal rim	Lateral	Medial	-
Renal sinus	Not involved	Involved	-
Urinary collecting system	Not involved	Dislocated/infiltrated	-
Tumor diameter	≤ 4.0 cm	4.1-7.0 cm	> 7.0 cm

* 4-6 points = low risk; 7-9 points = intermediate risk; > 10 points = high risk.

Source: Adapted from Ficarra et al.^(12)^.

Studies have addressed the PADUA classification system and have shown it to be comparable to the RENAL score, especially regarding warm ischemia time and postoperative complications^(22,23)^. Draeger et al.^(22)^ evaluated more than 200 individuals who underwent open NSS and reviewed their PADUA scores. The authors found that the score correlated significantly with longer operative time and longer warm ischemia time. On the basis of the PADUA score, renal masses were stratified into two groups (< 8 and ≥ 8 points), and there was a significant difference between the two in terms of the severity of complications, which was greater in the ≥ 8 point group. Tyritzis et al.^(24)^ calculated the PADUA scores for 74 patients who were submitted to open NSS. In a multivariate analysis, the authors found the PADUA score to be an independent predictor of complications, a score ≥ 8 being associated with a nearly 20 times higher risk of postoperative complications.

As with the RENAL nephrometry score, there are issues involving the application of the PADUA classification system. Some aspects of a renal mass, such as its proximity to the renal collecting system and renal sinus involvement, are dependent on the interpretation of the radiologist. Again, the polar lines should be determined in imaging reconstructions based on the renal axis in a sagittal view (Figure 2).

**Figure 4:**
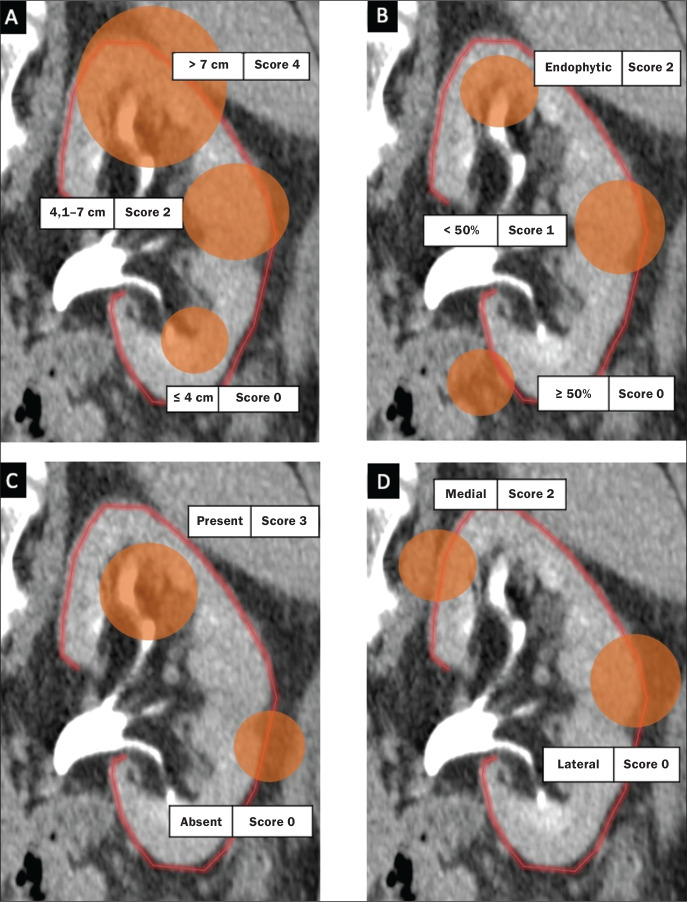
Reconstruction of a sagittal CT scan, showing the tumor features used in order to calculate the SPARE score. A: Tumor size. B: Exophytic proportion. C: Renal sinus involvement. D: Tumor location.

Source: Adapted from Huang et al.^(27)^.

One disadvantage of the PADUA classification system is the quantity of items to be evaluated (seven). That has led some authors to suggest that it is too complicated to use on a daily basis and, therefore, to gain popularity among professionals dealing with renal masses^(25)^. Consequently, Ficarra et al.^(26)^ developed the Simplified PADUA REnal (SPARE) nephrometry scoring system, excluding the variables that had not been found to be statistically significant and adding the contact surface area parameter, to see if it could provide greater accuracy in comparison with the original score. In a retrospective study, the authors evaluated 531 patients submitted to NSS and found that the SPARE score, which includes kidney location, renal sinus involvement, exophytic rate, and tumor size (Figure 4), showed performance comparable to that of the original PADUA score. Adding contact surface area to the original score did not increase the accuracy of either score to predict overall complications. The authors concluded that the new version of their score could replace the old one, especially because it evaluates fewer items without compromising the results.

The SPARE score was validated by Huang et al.^(27)^ in a retrospective study of 207 patients who had undergone NSS. The authors compared the RENAL, PADUA, and SPARE nephrometry scoring systems in terms of their ability to predict surgical outcomes. On the basis of the SPARE score, the authors stratified the patients into low-, intermediate-, and high-risk groups (n = 120, n = 74, and n = 13, respectively). They detected significant differences among the three groups in terms of the complication rate, warm ischemia time, and percent change in renal function. Those authors found that the SPARE score correlated significantly with surgical outcomes, showing fair ability to predict favorable surgical outcomes. They also found that the SPARE nephrometry scoring system produces results comparable to those obtained with other nephrometry scoring systems in predicting adverse outcomes after NSS.

### C index

The C index, proposed by Simmons et al. in 2010^(13)^, is based on the pattern of tumor growth into the renal parenchyma and uses tumor centrality as the main parameter to be evaluated. A mid-polar reference point, corresponding to the center of the kidney, is assigned. Images are scrolled to identify sections showing the uppermost and lowermost borders of the kidney at the last section in which the kidney is seen on the CT scan. At the mid-section, a central reference point is assigned at the center of an imaginary ellipse drawn around the kidney periphery. Then the section with the largest tumor diameter is identified. A 90° vertical axis digital reference point is assigned to all images in CT imaging software. The Pythagorean theorem is used in order to calculate the distance from the center of the tumor to the center of the kidney.

The C index classification system was validated in a retrospective study of patients who underwent laparoscopic NSS^(28)^. This system has been criticized because of the complexity of the formulas and measurements^(28)^, as well as because it evaluates only the size and depth of penetration into the renal parenchyma^(25,27)^.

Samplaski et al.^(29)^ reported that the C index is associated with the postoperative nadir of the estimated glomerular filtration rate (eGFR), the percent decrease, and the eGFR after NSS. The authors found that a C index ≤ 2.5, in comparison with a C index > 2.5, resulted in a 2.2-times greater risk of a ≥ 30% decrease in the postoperative eGFR.

### NePhRO

The NePhRO scoring system is an evolution of the RENAL nephrometry score^(14)^, based on four components related to tumor characteristics and kidney anatomy. It divides the kidney and the masses into anatomical zones, avoiding numbers, letters, and lines that can result in observer-dependent variations. The NePhRO scoring system is easier to learn than is the RENAL nephrometry score, and the results provided by the former are more easily understood^(14,25)^.

The two first NePhRO components are related to anatomical zones of the kidney, and the other two are tumor characteristics that aid in the classification. Each of the components are scored from one to three. After the four components have been defined, individual scores are established and summed. The final score indicates whether a mass is of low, intermediate, or high complexity.

**Figure 5:**
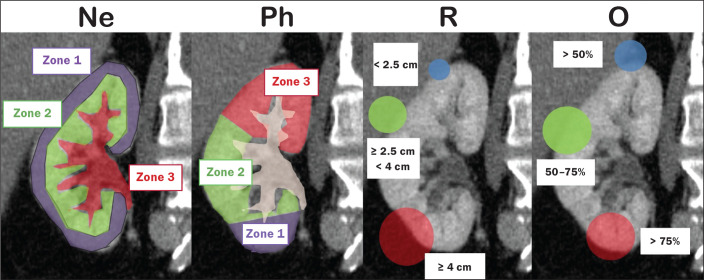
Sagittal CT scan of the kidney showing the renal zones and tumor characteristics used in order to calculate risk with the NePhRO score. Ne, proximity to the renal collecting system; Ph, physical zones; R, radius of the mass; O, organization of the mass; Zone 1, the lower pole, below the renal collecting system; Zone 2, lateral to the collecting system without touching it and excluding the upper pole; Zone 3, the upper pole, the collecting system, and all components of the hilum.

Source: Adapted from Hakky et al.^(14)^.

The first NePhRO component evaluated is the proximity of the mass to the renal collecting system, which is defined as the distance (in cm) between the mass and the renal parenchyma or as contact between the mass and the collecting system. Each segment is designated as a zone, and the points are assigned for each zone. As depicted in Figure 5 and described in Table 3, a mass is given one point if it is restricted to the renal cortex, two points if it has invaded the medulla, and three points if it is in contact with the collecting system.

**Table 3:** Renal features employed in order to calculate the NePhRO score.

Feature	Zone 1	Zone 2	Zone 3
Nearness	Mass in contact with the cortex	Mass in contact with the medulla	Mass in contact with the renal collecting system or crossing the renal sinus
Physical zones	Lower pole below collecting system	Lateral but not touching collecting system	Upper pole or touches collecting system
Radius (diameter)	< 2.5 cm	≥ 2.5 cm but < 4.0 cm	≥ 4 cm
Organization	> 50% exophytic	50-75% endophytic	> 75% endophytic
Points^*^	1	2	3

Zone 1, the lower pole, below the renal collecting system; Zone 2, lateral to the collecting system without touching it and excluding the upper pole; Zone 3, the upper pole, the collecting system, and all components of the hilum.

* 4-6 points = low risk; 7-9 points = intermediate risk; > 10 points = high risk.

Source: Adapted from Hakky et al.^(14)^.

The second NePhRO component evaluated is the physical location of the mass in relation to the anatomical zones of the kidney. The kidney is divided in three zones: zone 1 is the lower pole, below the renal collecting system, and the polar lines have to be drawn; zone 2 is lateral to the collecting system without touching it and excludes the upper pole; and zone 3 encompasses the upper pole, the collecting system, and all components of the hilum. A mass is given one point if it is in zone 1, two points if it is in zone 2, and three points if it is in zone 3.

The third NePhRO component evaluated is the diameter of the mass (in centimeters). A mass is given one point if it has a diameter of less than 2.5 cm, two points if it has a diameter equal to or greater than 2.5 cm but less than 4 cm, and three points if it has a diameter of 4 cm or more.

The fourth NePhRO component evaluated is based on anatomical zones that qualify the organization of the mass. A mass is given one point if more than 50% of its growth is exophytic, two points if 50-75% of its growth is endophytic and it is located in zone 2, and three points if more than 75% of its growth is endophytic or it is located in zone 3.

The scores for each NePhRO component are summed, and the total score is used in order to stratify masses by risk. As illustrated in Figure 5 and detailed in Table 3, the NePhRO scoring system stratifies risk as low (4-6 points), moderate (7-9 points), and high (10-12 points).

In a retrospective study published in 2014, Hakky et al.^(14)^ evaluated the NePhRO scoring system in 166 patients who had undergone NSS. The authors found that the NePhRO score was predictive of all 37 of the perioperative complications observed. In a univariate analysis, they also found that clinical stage, intraoperative blood loss, and tumor diameter correlated with the NePhRO score. In a subsequent study, Kriegmair et al.^(30)^ applied univariate and multivariate logistic regression to evaluate the utility of the NePhRO scoring system in 200 patients submitted to open NSS. Those authors found that a higher NePhRO score was significantly associated with complications such as longer warm ischemia time, a decrease in the eGFR, and opening of the collecting system. They also found that age and body mass index were independent factors associated with adverse outcomes.

### DISCUSSION

In candidates for NSS, there is a set of characteristics that must be considered before the treatment can be indicated. Those characteristics include age, comorbidities, body mass index, and the imaging aspects of the renal mass^(31)^.

Nephrometry has improved the fidelity of reports and comparability across studies evaluating the management of renal masses. In previous studies using nephrometry to describe such masses, terms such as central, hilar, and endophytic/exophytic have predominated. To gain popularity among urologists and radiologists, a nephrometry scoring system must show good results in predicting adverse outcomes and should be easily understood in order to be applied preoperatively in every case.

The radiological evaluation of renal masses is still a matter of discussion among radiologists. Although the majority of lesions can be easily detected and correctly characterized, misdiagnoses may occur and are often related to methodological limitations, inappropriate imaging protocols, or misinterpretation^(15)^. Because all nephrometry scoring systems depend on radiological evaluation, a detailed scan must be performed to accurately identify the characteristics of the mass and its relationship with the renal structures.

New imaging techniques allow acquisition of thin-slice images during a short breath hold, thus minimizing artifacts. An appropriate protocol for the detection of renal lesions should focus not only on optimizing multiphase acquisition but also on narrow collimation and reduced pitch, and multiplanar reconstructions of thin-slice images should be routinely performed^(32)^. That is important, because the kidney is anatomically positioned on an angular axis in relation to the spine. Therefore, if the reconstruction is based on the spinal axis, the localization of the mass in relation to the kidney can be inaccurate and polar lesions can be difficult to identify.

Compared with CT, MRI has better resolution and has the advantage of not exposing patients to ionizing radiation^(33)^. When the CT findings are inconclusive, the patient can be better evaluated by an MRI scan, which can also be an option in patients for whom the use of intravenous contrast is contraindicated^(34,35)^. In that scenario, aspects such as proximity to the renal collecting system may be evaluated by using urine, rather than intravenous contrast, as the contrast medium.

**Figure 6:**
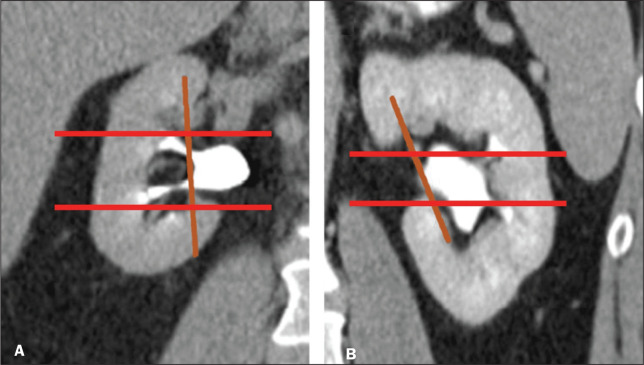
Contrast-enhanced coronal CT scans of the kidney, in the delayed phase, of two different patients, showing two types of renal pelvis: extrarenal (A) and intrarenal (B). Polar lines (red lines) drawn on each image, demonstrating the difference between the types of renal pelvis.

The imaginary polar lines may also cause misinterpretation and introduce bias during a radiological evaluation. That can happen when there is a large extrarenal pelvis that distorts the renal collecting system. In addition, when a physician draws the imaginary polar lines to apply a nephrometry scoring system, those lines could be above or below their correct locations, leading to an evaluation error and, in some cases, influencing the surgical outcome regarding urine leakage after NSS (Figure 6).

Nephrometry scoring systems consider only the aspects of the masses, neglecting one feature relevant to planning an NSS: the individual characteristics of the patient. Factors such as perirenal fat, age, previous surgery, anatomy, and an underlying disease may adversely influence the result of the procedure^(7,23,30,31)^. For example, a thin young patient with no comorbidities presenting with a 3.0-cm exophytic mass located in the lower pole of the left kidney, which would be categorized as low risk in any nephrometry scoring system, can represent a case that is technically less demanding than is that of an obese patient with a history of cholecystectomy and presenting with a 2.0-cm mass that is 50% endophytic and located in the posterior upper pole of the right kidney, which would also be categorized as low risk in any nephrometry scoring system.

**Figure 7:**
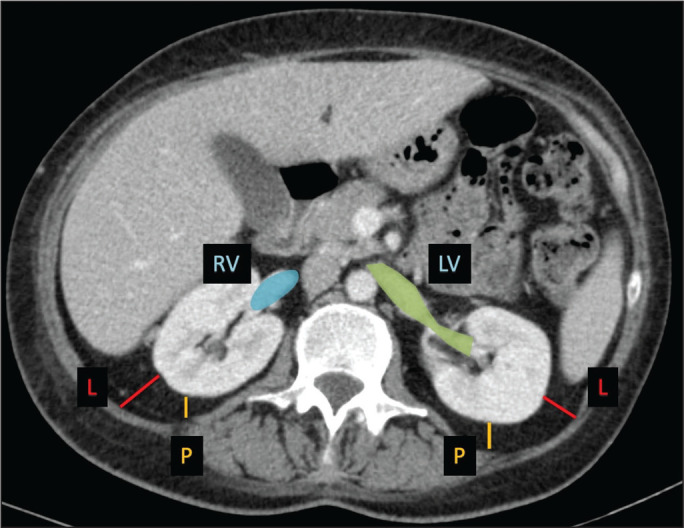
Axial CT scan demonstrating the method of measuring perinephric fat at the level of the renal vein to determine the MAPS. RV, right (renal) vein; LV, left (renal) vein; L, lateral; P, posterior.

Source: Adapted from Davidiuk et al.^(36)^.

Davidiuk et al.^(36)^ classified perirenal fat according to the CT findings in patients who were candidates for NSS, creating an image-based nephrometry scoring system known as the Mayo Adhesive Probability Score (MAPS). Because the perirenal fat is attached to the renal capsule, it may cause significant bleeding when removed during the dissection of a tumor, potentially resulting in perforation of the parenchyma or of the tumor. The method of measuring perirenal fat at the level of the renal vein is depicted in Figure 7. To determine the MAPS, perirenal fat is graded according to the presence of stranding (Table 4). If the fat around the kidney has no stranding, zero points are given; if it has some dense stranding but no thick bars of inflammation, it is designated grade 1 and two points are given; and if it has severe stranding and thick image-dense bars or lines of inflammation, it is designated grade 2 and three points are given.

**Table 4:** Grading of perirenal fat stranding to determine the MMAPS.

Grade	Description	Points
0	Fat around the kidney has no stranding	0
1 (mild/moderate)	Fat around the kidney has some image-dense stranding but no thick bars of inflammation	2
2 (severe)	Fat around the kidney has severe stranding with thick image-dense bars of inflammation	3

Source: Adapted from Davidiuk et al.^(36)^.

Spaliviero et al.^(37)^ recently proposed the Arterial Based Complexity nephrometry scoring system. The authors evaluated 179 patients who underwent NSS and studied the order of vessels transected or dissected during the procedure. Different scores were assigned to tumors requiring transection of interlobular arteries, arcuate arteries, segmental arteries, or arteries in close proximity to the renal hilum. They found that the reader score assignments correlated significantly across all reader pairs and concluded that the Arterial Based Complexity scoring system for NSS is intuitive, is easy to use, and correlates well with perioperative morbidity.

A number of studies comparing the various nephrometry scoring systems have shown that they are all similar in terms of their ability to predict adverse outcomes in NSS, despite having some limitations, especially regarding their applicability^(23,25,28,38,39)^. The use of complex formulas and the evaluation of multiple aspects of masses can make it difficult for a nephrometry scoring system to be accepted by the radiology and urology communities. That makes the RENAL nephrometry score slightly superior to the others in terms of acceptance^(39)^. In addition, most cases that are considered highly complex in any nephrometry scoring system are submitted to radical nephrectomy rather than NSS, making it difficult to evaluate a significant number of high-score cases^(20,25)^.

Some relevant aspects related to nephrometry scoring systems that are difficult to take into consideration are the experience and skill of the surgeon performing an NSS, as well as how a specific case will be approached. All nephrometry scoring systems have been based on the retrospective evaluation of laparoscopic NSS, although some have also been based on the retrospective evaluation of open NSS, which is not the current reality, because most open NSS procedures, especially difficult ones, are converted to robotic NSS^(6-8)^. A tumor resection that may be difficult for one surgeon may be feasible for a more experienced one.

Another important aspect is the correlation among CT scans, MRI scans, and three-dimensional (3D) reconstructions, in terms of the nephrometry score. Campos et al.^(40)^ analyzed the readings and RENAL nephrometry scores assigned by radiologists and urologists on the basis of each image type. The authors found that, in 37.5% of the patients evaluated, there was a statistically significant difference in the RENAL nephrometry score between the simple images and the 3D reconstructions, which could affect the management of cases by changing their eligibility for NSS. The authors also found that the use of 3D reconstructions in cases of intermediate complexity has a statistically significant impact on the perception of the RENAL nephrometry score, which plays an important role in surgical planning, whereas it was less relevant in cases of low and high complexity. The use of 3D reconstructions to determine the nephrometry score facilitates the work of surgeons by informing decisions regarding surgical planning, including selective vascular planning, as well as allowing difficulties to be anticipated and complications to be predicted, thus increasing the success rate of partial nephrectomy, with greater nephron preservation, which translates to better renal function^(40)^.

## CONCLUSION

The use of nephrometry scores for tumors of any degree of complexity (based on 3D reconstructions for those of intermediate complexity) now plays a major role in predicting adverse outcomes in NSS. The urology and radiology communities should decide which nephrometry scoring system will prevail and be used in daily practice. There is a need for further studies comparing the various nephrometry scoring systems as a means of evaluating cases prior to robotic NSS.
